# Predicting clinical outcomes in chordoma patients receiving immunotherapy: a comparison between volumetric segmentation and RECIST

**DOI:** 10.1186/s12885-016-2699-x

**Published:** 2016-08-23

**Authors:** Kathleen E. Fenerty, Les R. Folio, Nicholas J. Patronas, Jennifer L. Marté, James L. Gulley, Christopher R. Heery

**Affiliations:** 1 Laboratory of Tumor Immunology and Biology, Center for Cancer Research, National Cancer Institute, National Institutes of Health, 10 Center Drive, Room 13N208, Bethesda, MD 20892 USA; 2 Cancer Imaging Program, Division of Cancer Treatment and Diagnosis, National Cancer Institute, National Institutes of Health, Bethesda, MD USA; 3 Genitourinary Malignancies Branch, National Cancer Institute, National Institutes of Health, Bethesda, MD USA

**Keywords:** Chordoma, Volumetric, RECIST, Radiologic, Response criteria

## Abstract

**Background:**

The Response Evaluation Criteria in Solid Tumors (RECIST) are the current standard for evaluating disease progression or therapy response in patients with solid tumors. RECIST 1.1 calls for axial, longest-diameter (or perpendicular short axis of lymph nodes) measurements of a maximum of five tumors, which limits clinicians’ ability to adequately measure disease burden, especially in patients with irregularly shaped tumors. This is especially problematic in chordoma, a disease for which RECIST does not always adequately capture disease burden because chordoma tumors are typically irregularly shaped and slow-growing. Furthermore, primary chordoma tumors tend to be adjacent to vital structures in the skull or sacrum that, when compressed, lead to significant clinical consequences.

**Methods:**

Volumetric segmentation is a newer technology that allows tumor burden to be measured in three dimensions on either MR or CT. Here, we compared the ability of RECIST measurements and tumor volumes to predict clinical outcomes in a cohort of 21 chordoma patients receiving immunotherapy.

**Results:**

There was a significant difference in radiologic time to progression Kaplan-Meier curves between clinical outcome groups using volumetric segmentation (*P* = 0.012) but not RECIST (*P* = 0.38). In several cases, changes in volume were earlier and more sensitive reflections of clinical status.

**Conclusion:**

RECIST is a useful evaluation method when obvious changes are occurring in patients with chordoma. However, in many cases, RECIST does not detect small changes, and volumetric assessment was capable of detecting changes and predicting clinical outcome earlier than RECIST. Although this study was small and retrospective, we believe our results warrant further research in this area.

## Background

The Response Evaluation Criteria in Solid Tumors (RECIST) are the current standard for measuring treatment response in patients with malignant solid tumors [1]. However, RECIST has many limitations. RECIST 1.1, which calls for measurement of the longest diameter of the tumor (or perpendicular short axis of malignant lymph nodes), does not adequately represent the size of nonspherical lesions, nor does it reflect anisotropic changes in tumor size. Furthermore, it accounts for only five tumors per patient, with a maximum of two tumors per organ system.

The advent of advanced segmentation capabilities in PACS (Picture Archiving Communications Systems) on CT and MR has made volumetric segmentation an increasingly common alternative to RECIST. Segmentation consists of object recognition and delineation for the purpose of extracting quantitative information, such as tumor volume [2] and density [3]. It has many advantages over one-dimensional RECIST measurements, including the capability to assess all measureable lesions instead of just five lesions per patient. This has been shown to decrease variance in assessment of tumor burden [4]. Volumes have also been shown to demonstrate more consistency than linear measurements in phantoms (specially designed objects that are scanned to evaluate imaging technology) [5] and in retrospective studies [6]. It is thought that volumes better reflect actual changes in tumor size [7] and better reflect clinical outcomes [8]. Volumetric segmentation has also been shown to be reproducible, even in complex intracranial tumors [9].

Volumetric assessments may be particularly useful in certain tumor types. Chordoma is a rare, slow-growing neoplasm that arises from the remnants of the notochord. Many challenges are associated with chordoma patient care and research. Because it is a rare disease, literature about chordoma is scarce. Although it is commonly assumed that chordoma does not metastasize often, recent studies have indicated that it metastasizes more than previously thought [10]. For this reason, clinicians may not look for metastases or may fail to identify them because metastatic lesions can look like benign cysts, particularly in the liver [10].

RECIST is especially inadequate for evaluating chordoma tumor burden because lesions are generally lobulated and heterogeneous. Furthermore, changes in tumor size may not be readily detectable by RECIST because of chordoma tumors’ typically slow growth; however, owing to the proximity of these tumors to vital structures in the sacrum and clivus, small changes in size have significant clinical consequences. The urgent need for improved methods of assessing tumor burden in chordoma make this disease a good candidate for a volumetric segmentation study.

## Methods

### Patients

Our cohort consisted of 21 chordoma patients from two ongoing National Cancer Institute Institutional Review Board (IRB)–approved phase I clinical trials of therapeutic cancer vaccines. Eleven patients received the yeast-brachyury vaccine GI-6301 (NCT01519817) [11]. Thirteen received MVA-brachyury-TRICOM vaccine (NCT02179515), three of whom had previously received the yeast-brachyury vaccine. CT and MR scans were acquired at baseline and during treatment, and patients who went off trial continued to have follow-up scans. Patients had 2–14 appointments at which imaging was done (median, five). Although all patients had surgery and/or radiation, these treatments were most often given before the baseline scans. Volumetric segmentations were done on subsequent scans up through the most recent scan available for each patient. One patient had to be re-baselined after an ablation for the purpose of our analysis. As a result, that case is used as two separate data points (pre- and post-ablation) for radiologic time to progression (TTP) analysis. Two patients were excluded for not having at least two time points with CT and MR, and another patient was excluded because symptoms recurred after stopping steroids to enroll on a clinical trial. This resulted in three patients who were not included in the Kaplan-Meier analysis and one patient who had two data sets, totaling 19 evaluations. The two patients without follow-up scans were still included in other analyses for the purpose of assessing resources required for volumetric segmentation [12]. This research was conducted on images collected during two clinical trials, which were run in compliance with the Helsinki Declaration and were approved by the Center for Cancer Research, National Cancer Institute Institutional Review Board.

### Imaging

CT scans of the chest, abdomen, and pelvis were acquired at baseline (pretreatment) and at 8- to 12-week intervals following treatment initiation using any of the following scanners: Siemens Definition, Biograph, or Flash (Siemens Healthcare USA, Malvern, PA), Toshiba Aquilion ONE™ ViSION CT (Toshiba Medical Systems Corp., Tochigi, Japan), or GE Lightspeed (GE Medical Systems, Waukesha, WI).

Patients received contrast-enhanced CT scans using 0.6- to 2.5-mm collimation, 120 kVp, 150–240 reference mAs (with dose modulation), and 0.25- to 0.75-sec rotation time. Images were pushed to our PACS as contiguous 5 × 5-mm and 2 × 1-mm overlap axial slices for volumetric assessments and reformats (e.g., coronal). Scans were obtained with patients coached to full inspiration, supine from chest to pelvis in one acquisition, and with weight-based (2 mg/kg) i.v. contrast (Isovue 300 at 2 mL/sec) after a 70-sec delay.

One of the following scanners was used to obtain MR scans: 3 Tesla (3 T) Verio (Siemens), 3 T Achieva TX (Philips Healthcare, Andover, MA), 1.5 T Aera (Siemens), 3 T mMR (Siemens), or 1.5 T Achieva (Philips). Patients received TSE T1 axial and coronal imaging, TSE T2 axial and coronal imaging with fat suppression (or STIR), and axial diffusion-weighted imaging with B values of 0, 250, and 800. Apparent diffusion coefficient maps were generated from the 0 and 800 B values. All precontrasted images were acquired at a slice thickness and imaging gap of 6 × 2 mm.

Prior to contrast administration, a precontrast 3D Axial T1-weighted sequence (3-mm overlapping VIBE/DIXON/or E-Thrive) was obtained in a breath-held fashion. Following injection of i.v. gadolinium-based contrast (0.2 mL/kg, injected at 2 mL/s) (Magnevist®, Schering AG, Berlin, Germany and MultiHance®, Bracco, Milan, Italy), postcontrast images were obtained in identical fashion as the precontrast 3D images. Image acquisition time points were 20 sec, 70 sec, and a 3-min delay. All data were automatically subtracted from the precontrast acquisition. A final postcontrast 3D T1-weighted coronal image (3-mm overlapping VIBE/DIXON/or E-Thrive) was obtained at the conclusion of the MR examination.

### RECIST measurement

Tumors were evaluated using RECIST 1.1 guidelines [1], which call for one-dimensional, longest-diameter measurements in the axial plane. A maximum of five lesions may be evaluated in each patient, with no more than two per organ system.

### Volumetric measurement

A neuroradiologist (NP) reviewed the MR sequences to determine the best ones to use for segmentation. Post-contrast scans were not as useful as expected due to prior radiation and surgical treatments; enhancement was poor and tumors could not be differentiated from adjacent structures. Fat-suppressed T2-weighted and STIR sequences were deemed the most appropriate for segmenting sacral and paraspinous tumors, whereas post-contrast FLAIR sequences were used for clival lesions. Contrast-enhanced CT sequences were used to segment all metastases.

A research assistant (KF) performed the segmentations using the lesion management application within PACS (Vue PACS v 12.0, Carestream Health, Rochester, NY) as previously described [12]. In short, the proprietary software allows the user to identify the edges of the lesion with a digital caliper-like tool and then, based on imaging characteristics, the software generates a proposed border for the lesion across all cuts. To do this, the Vue PACS livewire segmentation tool applies a combination of fast marching [13] and level set [14] algorithms together with shape interpolation for region growing. The cost functions are based on image gradient strengths and image intensity histograms in order to determine the expansion limits. The user (KF) can then correct the border with a correction tool. MR was used for segmenting primary tumors and CT for metastatic disease. Bone metastases were not evaluable by volumetric segmentation. Tumors with long diameters < 0.5 cm were deemed immeasurable due to the inherent variability created by measuring very small lesions, similar to what is outlined in RECIST 1.1. Masses were reviewed and deemed to be measurable tumors based on clinical assessment and imaging characteristics; not all were biopsy-confirmed.

### Radiologist review

A neuroradiologist with 30 years of experience (NP) validated volumetric segmentations of primary tumors, and a body radiologist with 20 years of experience (LF) validated segmented metastatic tumors.

### Comparison techniques/statistics

Using the following criteria, we divided patients into two groups independent from radiologic analysis for TTP. Patients were placed into either a good or a poor clinical outcome group, based on the presence (poor) of ≥ 1, or the absence (good) of all of the following clinical indicators: (1) increasing tumor-related pain requiring significant change in pain medications, (2) increasing neurologic dysfunction, and/or (3) decreasing ECOG performance status due to tumor-related symptoms [15]. The determination of clinical outcome was made retrospectively at least six months after initial imaging studies.

**Fig. 1:**
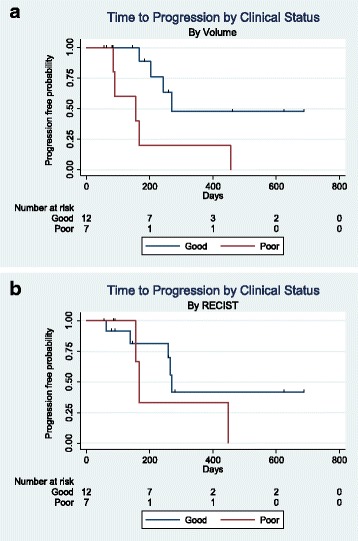
Volumetric assessment was superior to RECIST at predicting clinical status in this cohort. **a** Time to radiologic progression by volume. Actuarial median for good clinical outcome = 271 days; median for poor outcome = 156 days; *P* = 0.012; HR for good vs. poor clinical status = 0.21, *P* = 0.023. **b** Time to radiologic progression by RECIST. Actuarial median for good clinical outcome = 271 days; median for poor outcome = 167 days; *P* = 0.37; HR for good vs. poor clinical status = 0.52, *P* = 0.38

For patients in each category, Kaplan-Meier curves were used to calculate radiologic TTP by RECIST and by volume (Fig. 1) using the log-rank test for equality of survivor functions. A hazard ratio was also calculated using the Cox proportional hazard regression. TTP by RECIST was assessed using RECIST 1.1, with progressive disease (PD) being an increase of ≥ 20 % in the sum of the longest diameters (SLD). TTP by volume was determined based on previously outlined criteria [16], with PD being an increase of ≥ 40 %. TTP was assessed based on date of enrollment to time of PD by RECIST or volumetric criteria. Patient data were censored if PD criteria were not met on the last imaging studies prior to a local intervention on a target lesion or date of last available imaging.

## Results

### Patient demographics

Our cohort was 85.7 % male (18/21 patients) and had a median age of 60 (Table 1). Primary tumors were located in the spine, sacrum, or clivus, and all patients had been treated with surgery and/or radiation prior to baseline. Although only 66.7 % of patients (14/21) were diagnosed with metastatic disease at baseline, retrospective analysis identified two additional patients with small metastases at baseline.

**Table 1:** Patient demographics

Age range (median)	32–82 (60)
Gender	# (%)
Male	18 (85.7)
Female	3 (14.3)
Prior treatment	# (%)
Surgery	20 (95.2)
Radiation	20 (95.2)
Primary tumor	# (%)
Lumbar/sacral spine	14 (66.7)
Clivus	6 (28.6)
Cervical spine	1 (4.8)
Metastatic disease at baseline	# (%)
Yes	14 (66.7)
No	7 (33.3)

Most patients had 1–5 follow-up appointments (Table 2), although some had more, and two were excluded from our analysis for not having at least one follow-up CT and one follow-up MR that could be analyzed.

**Table 2:** Number of follow-up appointments with ≥ 1 CT or 1 MR/patient

# of follow-ups	# of patients (%)
1–5	14 (66.7)
6–10	5 (23.8)
11–15	2 (9.5)

*CT* computed tomography, *MR* magnetic resonance

A retrospective analysis found that 76.2 % of patients (16/21) had metastatic disease distributed throughout the lung, liver, lymph nodes, subcutaneous tissue, and other soft tissue (Table 3).

**Table 3:** Distribution of metastases at baseline and most recent follow-up

Location of metastases	# of patients (%)^a^ at baseline	# of patients (%)^a^ at most recent follow-up
Lung	9 (56.3)	10 (62.5)
Liver	9 (56.3)	9 (56.3)
Lymph nodes	2 (12.5)	5 (31.3)
Subcutaneous	4 (25.0)	4 (25.0)
Other soft tissue	5 (31.3)	6 (37.5)
Total # of patients with metastases	16 (100.0)	16 (100.0)

^a^Percentages based on 16 patients found to have metastases. Eleven patients (68.8 %) had bone metastases, which were not evaluable by volumetric segmentation. Values were obtained by retrospective analysis; thus two patients with unnoticeable metastatic disease at baseline are included

### Analysis of TTP

Patients in the good clinical outcome group (no poor clinical indicators, *n* = 12) appeared to have a longer TTP by volumetric assessment (*P* = 0.012, HR 0.21, *P* = 0.02) than patients in the poor clinical outcome group (≥1 poor clinical indicators; *n* = 7). However, there was no difference between the two groups by RECIST TTP analysis (*P* = 0.37, HR 0.52, *P* = 0.38).

### Case studies

Due to small sample size and extensive variability within our patient population, we found it useful to analyze a few case studies that exemplified instances in which volumetric assessment was useful and necessary, as well as instances in which RECIST provided sufficient information about tumor burden.

### Case 1

**Fig. 2:**
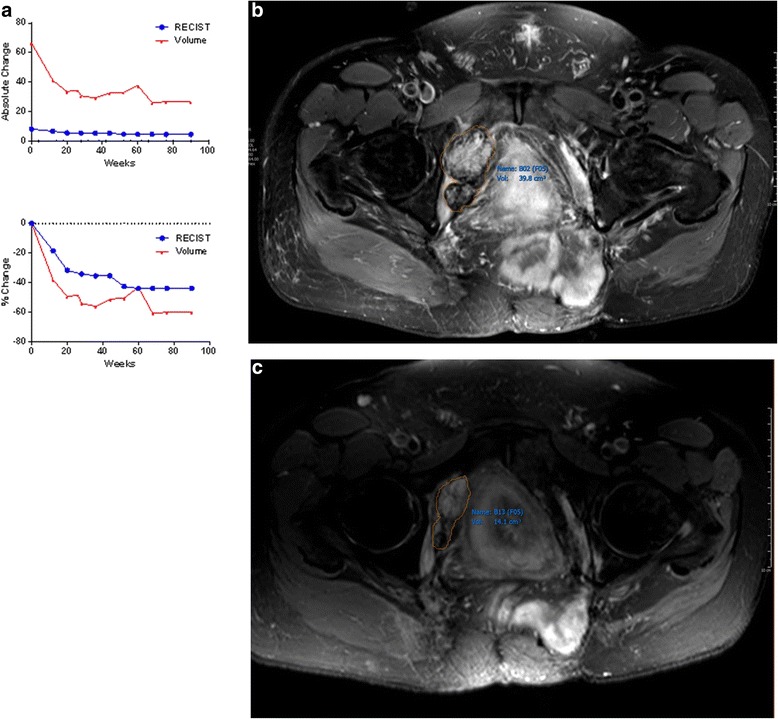
**a** Patient with a pelvic mass for whom RECIST is an adequate measure of tumor burden, and volumetric measurement provides little additional information. At the most recent follow-up appointment, RECIST indicated a partial response (–43.9 %) and volume indicated a minor response (–59.9 %). **b** Volumetric measurement of the patient’s primary lesion. **c** Six months later, the tumor had shrunk by both RECIST and volume

For patients who did very well or very poorly clinically, RECIST was often sufficient to illustrate the extent of their disease, while volumes provided little additional information. For one patient with recurrent pelvic masses who had done well clinically for over 2 years, RECIST indicated a partial response (–43.9 %) and volume indicated a minor response (–59.9 %). On July 8, 2013, the RECIST measurement for the patient’s primary lesion was 6.0 cm and its volume was 39.8 cm^3^. By January 6, 2015, the lesion measured 5.2 cm by RECIST and 14.1 cm^3^ by volume (Fig. 2). In cases where progression or response is less apparent, volumes may be superior to RECIST in predicting clinical outcome (see cases 2 to 4 below).

### Case 2

**Fig. 3:**
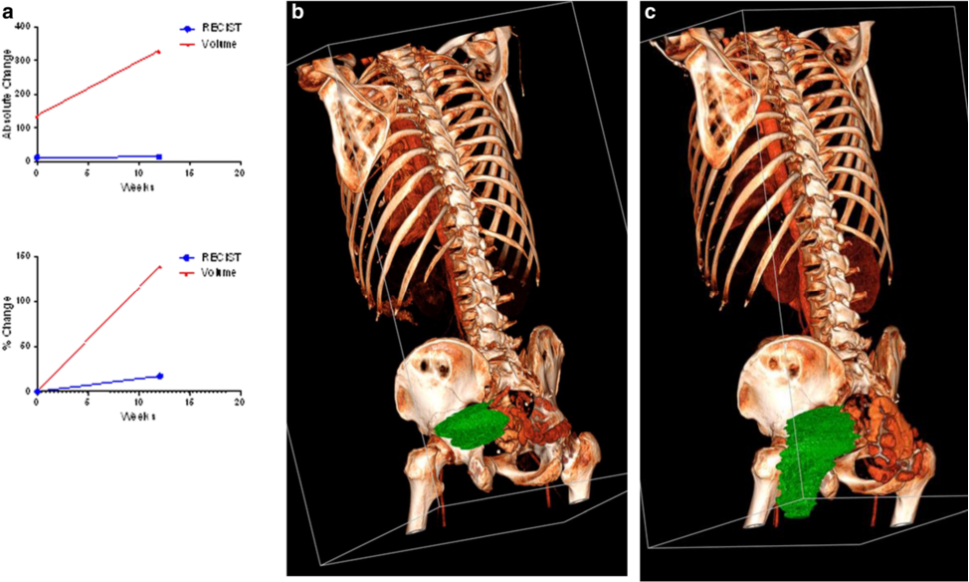
**a** Patient with a large presacral/pelvic mass had progressed by volume at 12-week follow-up (+139 %) but not by RECIST (+17.2 %). **b** 3D rendering of the patient’s lesion at baseline better illustrates tumor size and shape. **c** Three months later, the tumor had undergone drastic anisotropic growth not detectable by RECIST

One patient had severe pain that increased while on treatment and became difficult to manage by the restaging visit at 12 weeks. Based on that rapid progression of symptoms, the patient was retrospectively classified in the poor clinical outcome group. Axial measurement of the longest diameter of the presacral/pelvic tumor mass demonstrated growth that did not meet progression criteria (RECIST +17.2 %). However, the symptoms were consistent with significant tumor growth, which was observed volumetrically (+139 %), with the most notable growth in the cranial-caudal axis (Fig. 3).

### Case 3

**Fig. 4:**
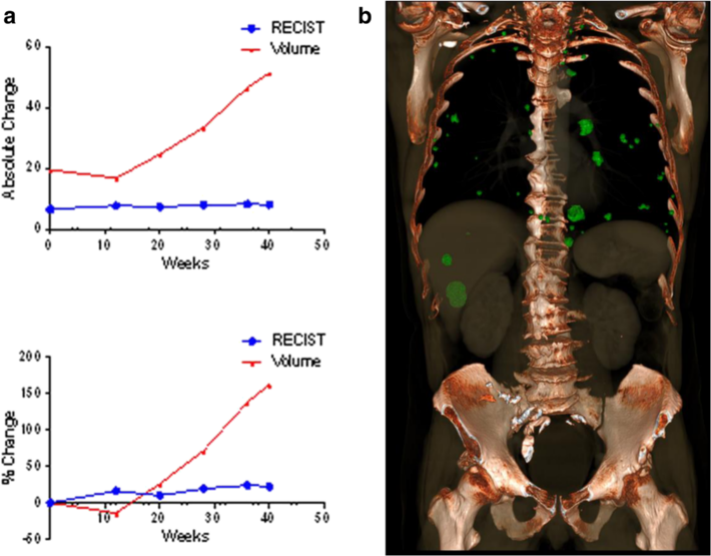
A case of metastatic disease to the liver and lungs in which volume indicated progressive disease earlier than RECIST. **a** PD was identified at 28-week follow-up by volume (+71.4 %), but not until 36-week follow-up by RECIST (+23.9 %). **b **A 3D rendering of this patient's tumors demonstrates the potential importance of measuring total tumor volume to determine treatment effect

In other patients, volume indicated PD earlier than RECIST. In one patient with metastatic disease to the liver and lungs, PD was identified at the 28-week follow-up by volume (+71.4 %), but not until the 36-week follow-up by RECIST (+23.9 %) (Fig. 4). In cases such as these, using total tumor volume as a metric of disease progression would allow patients to consider alternative therapies earlier.

### Case 4

**Fig. 5:**
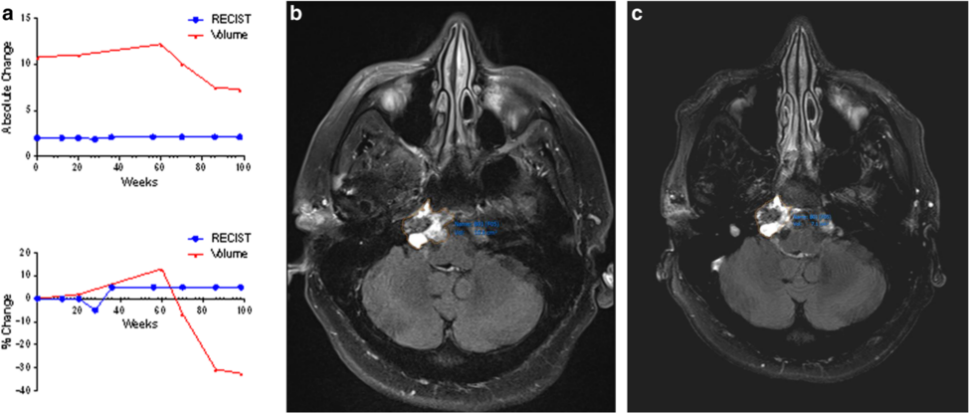
Patient with clival chordoma who seems to be experiencing clinical benefit from treatment. **a** At the most recent follow-up, volume is trending toward improvement (–32.4 %), whereas RECIST measurements have barely changed (+5.0 %). **b** The patient’s primary lesion. **c** Twenty-two months later, the patient’s RECIST measurement had not changed. The tumor was smaller by volume but had not yet reached a partial response by volumetric criteria

It can be difficult to determine the projected clinical course for a patient with chordoma for whom RECIST indicates almost no change over extended periods of time. For one patient with clival chordoma, RECIST showed an increase of 5 %, whereas volume indicated a decrease of 32.4 %. On July 9, 2013, the patient’s primary lesion measured 2.1 cm by RECIST and 10.6 cm^3^ by volume. By May 26, 2015, the lesion still measured 2.1 cm by RECIST but only 7.1 cm^3^ by volume (Fig. 5). The patient is doing well clinically, with subjective improvements in headaches related to the tumor mass.

## Discussion

Because our clinical experience indicated changes in symptoms prior to radiologic progression by RECIST, we sought to identify a tool that would give us an earlier insight into the trajectory of a given patient’s tumor. We noted a recurring pattern of tumor growth in imaged sections of tumors that were not those with the longest dimension. This finding led to the hypothesis that the total tumor volume may be changing despite lack of obvious change in the longest dimension, as measured by RECIST. Volumetric assessment of tumors has previously been difficult to perform. Radiation oncologists have used planning software to assess volume with promising results [17], but we wanted to determine if measuring volume could be more widely instituted. Recent improvements in imaging software have allowed for semi-automated assessment of total tumor volume. Volume assessment is still labor-intensive, but that limitation appears to be improving rapidly.

## Conclusions

In this hypothesis-generating study, we demonstrated the feasibility of using volumetric assessments and their potential impact on clinical decision-making. There were clear limitations to this study. First, we retrospectively applied a new, nonvalidated definition for clinical outcomes to a retrospective data set. Second, in this relatively small data set there was significant heterogeneity of tumor locations (sacral vs. spine vs. clival vs. metastatic disease). Third, the accuracy of measurements varied among our various imaging techniques. And finally, we used a 40 % cut-off for progression using volumetric assessment, which is not validated and was created based on different methods than we used in this study. These limitations preclude drawing definitive conclusions from the results. The use of clinical criteria retrospectively is perilous due to the possibility of bias influencing the outcomes. However, the clinical outcome groups were determined prior to the volumetric assessment and comparison to RECIST, limiting this concern. The heterogeneity of tumor locations is a major reason for the need to identify better imaging methods for chordoma and represents the nature of research in this rare disease. The potential variability between scans is an issue present in both RECIST measurement [18] and volumetric measurement, but is likely to be more pronounced on a single cut than over many cuts encompassing the entire tumor mass. Our choice of a cut-off for significant change by volume is based on a study evaluating volume by different methods, but we believe the rationale for this choice remains reasonable. Despite these weaknesses, the data presented here support the hypothesis that volumetric assessment may be a more sensitive tool for measuring early tumor progression in chordoma and suggest that further exploration of this method is worthwhile. For cases in which RECIST measurement demonstrates growth or regression, volumetric assessment is probably unnecessary and unlikely to have a significant impact. Volumetric assessment appears to be most useful in cases where RECIST can detect no discernable change in tumor size.

In chordoma management, small changes in tumor size may have significant clinical impacts due to the anatomic location of lesions. Cases 2, 3, and 4 presented here illustrate situations in which small changes in RECIST measurement may belie more significant growth in other dimensions. Patients were retrospectively grouped into good or poor clinical outcome categories based on relatively simple criteria. When we compared TTP in these groups by RECIST, we found no differences (*P* = 0.37, HR 0.52, *P* = 0.38). However, when we compared TTP by volumetric measurement, there was a clear separation of the curves (*P* = 0.012, HR 0.21, *P* = 0.02). While not definitive, these preliminary findings support our hypothesis that volumetric tumor assessment is a more sensitive tool for evaluating tumor growth in chordoma, and may be useful for predicting clinical outcomes in patients for whom RECIST demonstrates no change. Based on these findings, we suggest that clinical trials in chordoma should employ volumetric tumor assessment to determine the feasibility of real-time measurement and the potential impact on prospective clinical decisions.

## Abbreviations

CT: Computed tomography
ECOG: European Cooperative Oncology Group
HR: Hazard ratio
min: Minute
MR: Magnetic resonance
MVA: Modified vaccinia Ankara
PD: Progressive disease
RECIST: Response evaluation criteria in solid tumors
sec: Seconds
SLD: Sum of the longest dimensions
T: Tesla
TRICOM: Triad of costimulatory molecules
TSE: Turbo spin echo
TTP: Time to progression
